# Red Light Activation of Ru(II) Polypyridyl Prodrugs via Triplet-Triplet Annihilation Upconversion: Feasibility in Air and through Meat

**DOI:** 10.3390/molecules21111460

**Published:** 2016-11-01

**Authors:** Sven H. C. Askes, Michael S. Meijer, Tessel Bouwens, Iris Landman, Sylvestre Bonnet

**Affiliations:** Leiden Institute of Chemistry, Leiden University, Einsteinweg 55, Leiden 2333 CC, The Netherlands; s.h.c.askes@chem.leidenuniv.nl (S.H.C.A.); m.s.meijer@chem.leidenuniv.nl (M.S.M.); tessel.bouwens@student.uva.nl (T.B.); iris.landman@student.uva.nl (I.L.)

**Keywords:** photochemical upconversion, ruthenium prodrugs, phototherapy, liposomes, meat spectroscopy

## Abstract

Triplet-triplet annihilation upconversion (TTA-UC) is a promising photophysical tool to shift the activation wavelength of photopharmacological compounds to the red or near-infrared wavelength domain, in which light penetrates human tissue optimally. However, TTA-UC is sensitive to dioxygen, which quenches the triplet states needed for upconversion. Here, we demonstrate not only that the sensitivity of TTA-UC liposomes to dioxygen can be circumvented by adding antioxidants, but also that this strategy is compatible with the activation of ruthenium-based chemotherapeutic compounds. First, red-to-blue upconverting liposomes were functionalized with a blue-light sensitive, membrane-anchored ruthenium polypyridyl complex, and put in solution in presence of a cocktail of antioxidants composed of ascorbic acid and glutathione. Upon red light irradiation with a medical grade 630 nm PDT laser, enough blue light was produced by TTA-UC liposomes under air to efficiently trigger full activation of the Ru-based prodrug. Then, the blue light generated by TTA-UC liposomes under red light irradiation (630 nm, 0.57 W/cm^2^) through different thicknesses of pork or chicken meat was measured, showing that TTA-UC still occurred even beyond 10 mm of biological tissue. Overall, the rate of activation of the ruthenium compound in TTA-UC liposomes using either blue or red light (1.6 W/cm^2^) through 7 mm of pork fillet were found comparable, but the blue light caused significant tissue damage, whereas red light did not. Finally, full activation of the ruthenium prodrug in TTA-UC liposomes was obtained under red light irradiation through 7 mm of pork fillet, thereby underlining the in vivo applicability of the activation-by-upconversion strategy.

## 1. Introduction

Among the wide variety of photopharmacological compounds known to date, light-activatable ruthenium polypyridyl complexes have received considerable attention as they represent a promising family of anticancer prodrugs. In a process called “photoactivated chemotherapy” (PACT) [1,2,3,4,5], excitation with visible light transforms these non-toxic compounds into cytotoxic species via photosubstitution reactions. Like in photodynamic therapy (PDT), ruthenium-based PACT agents may greatly reduce undesired side effects for cancer patients by the excellent spatio-temporal control over activation. In addition, it is possible to anchor ruthenium complex to nanoparticles or liposomes, which allows for targeting the compound to tumors by making use of their leaky vasculature via the enhanced permeability and retention (EPR) effect [6,7]. For this class of photopharmacological compounds, the light-induced toxicity does not depend on the presence of oxygen, as opposed to photodynamic therapy (PDT) type 2, which functions by generating highly reactive oxygen species (ROS) [8,9]. Thus, using light-activatable Ru-complexes may be suitable for hypoxic tumor tissues for which PDT is not effective. One of the major challenges with ruthenium-based PACT agents is that they are mostly activated with blue or green light, which does not penetrate human tissues very well. Shifting the excitation wavelength of ruthenium complexes to the phototherapeutic window (600–950 nm) is necessary, but achieving such a shift by molecular design remains very difficult [10,11,12]. To circumvent this problem, upconverting drug delivery systems have been proposed that can not only transport the prodrug to the tumor, but also locally “upgrade” red or near-infrared photons into blue or green photons with which the prodrug is activated.

**Figure 1:**
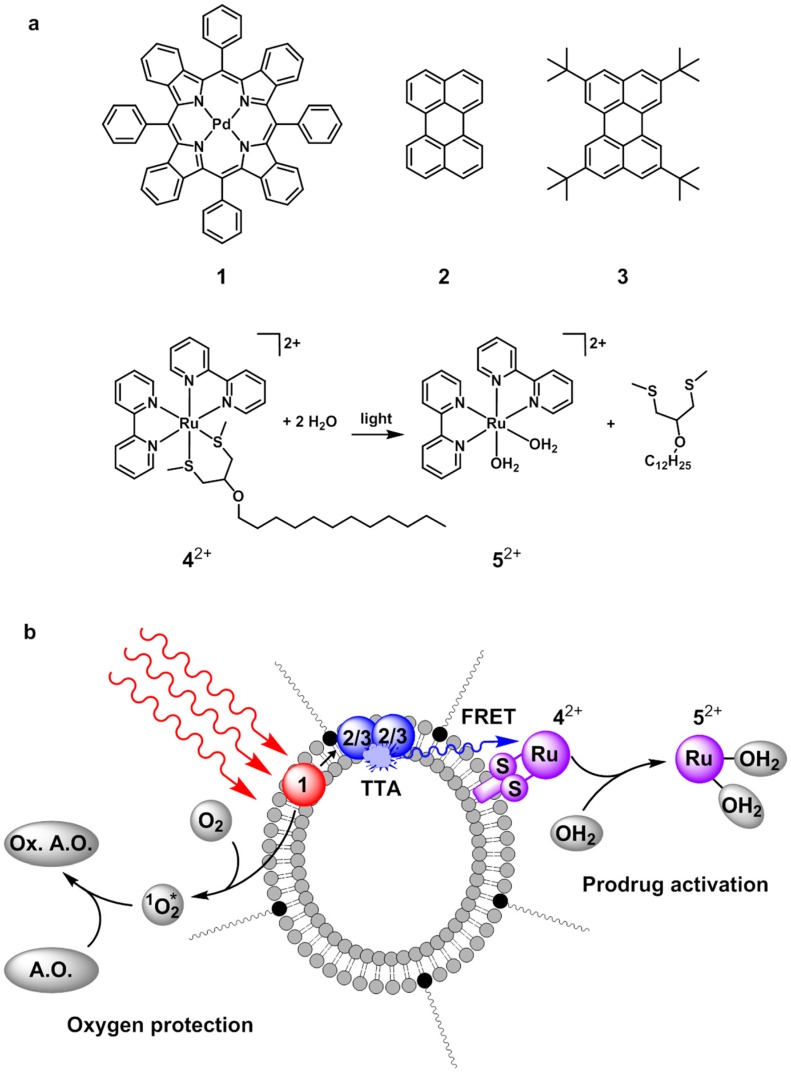
(**a**) Chemical structures of palladium(II) tetraphenyltetrabenzoporphyrin (**1**), perylene (**2**), 2,5,8,11-tetra(tert-butyl)perylene (**3**), Ru(II) compound **4**^2+^ and its photochemical conversion to **5**^2+^ upon visible light irradiation in presence of water; (**b**) Cartoon illustrating upconversion mediated Ru-prodrug activation in PEGylated liposomes in presence of antioxidants. Red-to-blue triplet-triplet annihilation (TTA) upconversion in the lipid bilayer is followed by non-radiative energy transfer (FRET) of the upconverted light to **4**^2+^, and subsequent photochemical reaction of **4**^2+^ to **5**^2+^. Meanwhile, an antioxidant (A.O.) protects the photochemical cascade from quenching by dioxygen by consuming the singlet-state oxygen (^1^O_2_*) that is initially generated upon irradiation of **1** until all oxygen is consumed. Hereby, the antioxidant becomes oxidized (Ox. A.O.).

Among the various methods available for performing light upconversion, triplet-triplet annihilation upconversion (TTA-UC) is one of the most advantageous for clinical application, because it requires low excitation intensity (down to 1 mW/cm^2^), employs sensitizers that have high molar absorptivity in the phototherapeutic window, and typically achieves upconversion quantum yields of 1%–5% also in aqueous solution [13,14,15]. TTA-UC is based on the photophysical interplay of photosensitizer and annihilator chromophores (see Figure S1) [16,17]. The photosensitizer absorbs low-energy light, after which intersystem crossing leads to a long-lived triplet state. This triplet state is transferred to the annihilator upon diffusional collision by means of triplet-triplet energy transfer (TTET); succession TTET events lead to a concentration buildup of long-lived triplet state annihilators molecules. Two triplet state annihilator molecules interact, resulting in triplet-triplet annihilation, in which one of them departs with all the energy of the pair and reaches a high-energy singlet excited state. Finally, this singlet excited state returns to the ground state by fluorescent emission of a high-energy photon, thereby realizing upconversion. TTA-UC has been demonstrated in various organic, inorganic, and/or supramolecular materials [15,18,19,20,21,22], as well as in nano- or micro-sized particles [23,24,25]. For biological applications, i.e., for drug delivery and activation [26,27] or bio-imaging [13,28,29,30,31,32,33], one of the main problems of TTA-UC is its sensitivity to molecular oxygen, which readily quenches the triplet state chromophores involved in the TTA-UC mechanism. To solve this issue, we and others recently showed that antioxidants such as l-histidine, ascorbic acid, or glutathione, can protect TTA-UC against oxygen: upon irradiation of TTA-UC vesicles in air, the photosensitizer initially generates singlet oxygen, which is scavenged by the antioxidant until all oxygen in the irradiated volume has been consumed (Figure 1b) [25,34,35,36,37]. Once the O_2_ concentration reaches a certain threshold, TTA-UC switches on and remains stable for minutes to even hours in spite of the air atmosphere.

Although our group previously demonstrated that blue-light sensitive Ru polypyridyl complex could be effectively activated by a red-to-blue TTA-UC in liposomes (Figure 1b), this demonstration was performed under argon, which is not relevant for clinical applications [26,27]. In the first part of this article, we report that it is possible to solve this issue by using the antioxidant strategy mentioned above. Ruthenium polypyridyl compounds such as **4**^2+^ (Figure 1a) are known [38,39] to photosubstitute their chelating bis-thioether ligand upon blue light irradiation to produce the cytotoxic species (Ru(bpy)_2_(OH_2_)_2_)^2+^ (**5**^2+^, Figure 1a) [2]. Usually, antioxidants have also good coordinating properties (e.g., histidine, glutathione, etc.), but our data demonstrate that these coordinating properties do not prevent the triggering of photosubstitution reactions in air for the liposome-bound ruthenium complex **4**^2+^ using red light and TTA-UC. In the second part of this article, the tissue depth at which TTA-UC is still able to generate blue light is evaluated by shining red light through increasing layers of pork and chicken meat. In the third and final part, we demonstrate not only that it is possible to fully activate the ruthenium prodrug through pork meat and using red light, but that it is also actually more advantageous to use red light instead of blue light, because there is less tissue damage.

## 2. Results and Discussion

### 2.1. Liposome Preparation

**Scheme 1:**
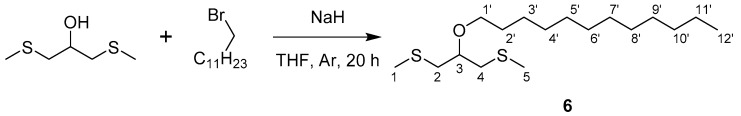
Synthesis of compound **6** from 1,3-bis(methylthio)-2-propanol and 1-bromododecane.

PEGylated upconverting liposomes containing or not the ruthenium compound **4**^2+^ (Scheme 1) were prepared by a standard hydration-extrusion protocol [26] in phosphate buffered saline (PBS). Where applicable (see below), before addition of PBS to the lipid film, the buffer was supplemented with a known concentration of l-ascorbic acid (l-Asc) and/or glutathione (GSH) as antioxidants, and neutralized to pH 7.0–7.6 with NaOH. For all samples the major component of the liposome membrane was a neutral phosphatidylcholine lipid, i.e., either 1,2-dilauroyl-*sn*-glycero-3-phosphocholine (DLPC, liposome samples are denoted “L” in Table 1) or 1,2-dimyristoyl-*sn*-glycero-3-phosphocholine (DMPC, liposomes are denoted “M” in Table 1). The liposomes were PEGylated with 4 mol % sodium *N*-(carbonyl-methoxy polyethyleneglycol-2000)-1,2-distearoyl-*sn-*glycero-3-phosphoethanolamine (DSPE-mPEG-2000), which is known to prevent aggregation and to prolong the lifetime of liposomes in the blood circulation [6,7]. Finally, the liposomes were doped with either the TTA-UC dye couple (0.05 mol % **1**, 0.5 mol % **2**), or all three components (0.05 mol % **1**, 1 mol % **3**, and 4 mol % **4**^2+^); Table 1 summarizes the exact formulation of all liposome samples prepared. All samples were characterized by dynamic light scattering (DLS), which showed that the hydrodynamic size (z-ave) of all liposomes varied from 130–170 nm with an average polydispersity index (PDI) of 0.1.

### 2.2. Anti-Oxidants Allow Efficient and Stable TTA-UC in Air with ***L12*** Liposomes

**Figure 2:**
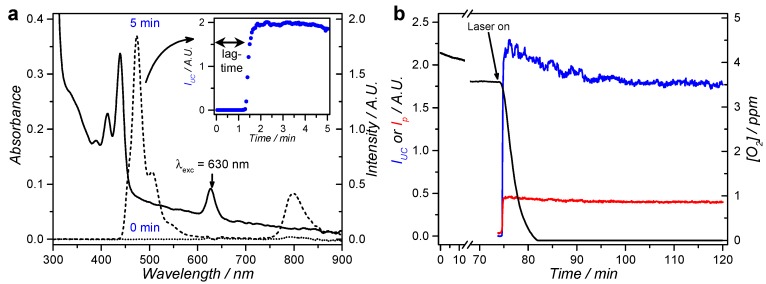
Emission spectroscopy in air of **L12** liposomes in PBS supplemented with l-Asc or GSH. (**a**) Absorption (solid) and emission spectra (dotted: *t* = 0; dashed: *t* = 5 min) of **L12** liposomes in PBS supplemented with 5 mM l-Asc. Inset shows the upconversion intensity (*I_UC_* at 474 nm) for the first 5 min of irradiation with 3 s intervals; (**b**) Time trace of the upconversion intensity (*I_UC_* at 474 nm, blue), phosphorescence intensity (*I_p_* at 800 nm, red), and dissolved oxygen concentration (black) during red light irradiation of **L12** liposomes in PBS supplemented with 5 mM l-Asc. The laser was turned on after 74 min in the dark, as indicated by the arrow. Exp. conditions: [DLPC] = 1 mM, [**1**] = 0.5 µM, [**2**] = 5 µM, 2.25 mL sample, T = 20 °C, 10 mW 630 nm laser excitation (80 mW/cm^2^) with approximately 8% of the volume simultaneously irradiated, pH 7.0–7.6.

Following our recently published strategy [25], the red-to-blue upconversion of DLPC liposomes **L12** was studied in presence of ascorbic acid or glutathione to investigate to which extent upconversion could occur in air. In this study, DLPC was used as main lipid because upconversion was found more efficient at room temperature in DLPC than in DMPC liposomes [40]. Thus, when **L12** was irradiated with red light (630 nm) at a power density of 80 mW/cm^2^ in air in presence of 5 mM l-Asc, emission spectra initially showed only weak phosphorescence of **1** at 800 nm and no upconversion emission (Figure 2a, inset). After a “lag time” of ~1 min irradiation, however, the phosphorescence at 800 nm suddenly intensified and intense upconversion emission at 474 nm was observed; both emission bands stabilized after 2 min (Figure 2a, inset). The experiment was repeated while probing the dissolved oxygen concentration (Figure 2b). Oxygen was consumed only very slowly in the dark, but was fully depleted after 5 min of red light irradiation. These results confirm that the antioxidant scavenges the singlet-oxygen that is generated by the photosensitizer upon irradiation [25,34]. When the experiment was repeated in presence of 0.25–50 mM l-Asc or 1–20 mM GSH, i.e., close to the biological concentrations of these two species [41], the “lag time” before upconversion progressively decreased with increasing antioxidant concentration to as low as 0.5 min lag time at 50 mM of l-Asc (Figure S2). It was also found that a “cocktail” containing both l-Asc and GSH synergistically minimized the lag-time: for a mixture of 1 mM l-Asc and 5 mM GSH the lag-time was 1.1 min, which was significantly shorter than that found with either of the antioxidants alone (3.2 min for 1 mM l-Asc and 13 min for 5 mM GSH). Therefore, a combination of these two antioxidants was used in all further experiments. From this first set of data, it appeared clearly that the addition of biologically relevant concentrations of antioxidants to upconverting liposomes results in stable red-to-blue TTA-UC in air.

### 2.3. Activation of a Photodissociative Ruthenium Complex Using Upconverting DMPC Liposomes in Air

**Figure 3:**
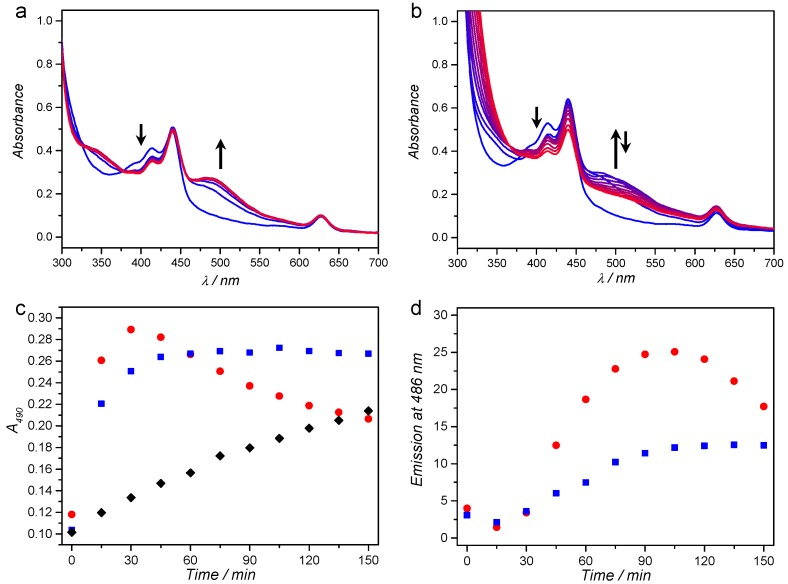
UV-vis absorption spectroscopy in 15 min intervals (gradient) during red-light irradiation of **M134** liposomes under: argon (**a**); and in air in presence of 10 mM l-Asc and GSH (**b**). Absorbance at 490 nm (**c**); and upconversion emission at 486 nm (**d**) as a function of time for the experiment under argon (blue squares) and in air in presence of antioxidants (red circles). As control, **M34** liposomes were irradiated under the same conditions under argon (**c**) (black diamonds). Conditions: 2 mL sample with 1 mM DMPC, irradiated at 37 °C with 150 mW 630 nm light (1.2 W/cm^2^). Arrows indicate the evolution of the spectrum in time at the indicated wavelengths (400 and 500 nm).

To investigate whether antioxidants could also protect the upconversion-mediated photoactivation of **4**^2+^ in air, **M134** liposomes were prepared at 1 mM bulk DMPC concentration that contained red-to-blue TTA-UC dye couple **1** and **3**, and the photodissociative ruthenium compound **4**^2+^. The photodissociation reaction with red light irradiation was performed in either deoxygenated conditions using argon bubbling for 30 min, or in air-equilibrated solution containing 10 mM l-Asc and 10 mM GSH. Both samples were irradiated at human body temperature (37 °C) with 630 nm light (150 mW, 1.2 W/cm^2^) for 2.5 h while recording the UV-vis absorbance (Figure 3a,b) and emission spectra every 15 min. The UV-vis spectrum at *t* = 0 shows the characteristic absorption bands of **1** at 440 and 630 nm, of **3** between 375 and 450 nm, while the absorbance of **4**^2+^ (Figure S3) is hidden under the bands of **1** and **3**. Under argon and in absence of antioxidants, the absorption band of the photoproduct **5**^2+^ evolves to completion within the first hour of irradiation, just as observed for complex **4**^2+^ in water upon blue light irradiation (Figure S4). By plotting the absorbance at 490 nm versus time (Figure 3c), it is clear that the photoreaction was completed after about 45 min of irradiation (40 µmol **4**^2+^ converted to **5**^2+^). While the photoreaction was progressing, the upconversion emission at 486 nm increased (Figure 3d) because complex **5**^2+^ dissociates from the membrane into the solution, thereby no longer quenching the emission of **3** via FRET (see Figure S3 for overlap between **3** and **4**^2+^) [26]. After 60–90 min of irradiation, both the absorption and emission spectra completely stabilized, showing that photosubstitution reaction was completed.

When red light irradiation was conducted in air and in presence of antioxidants, the same absorption band between 460 and 600 nm appeared in the first 30 min of irradiation (Figure 3b), which strongly resembled the absorption band of **5**^2+^. After that time-point however, this absorption band slowly disappeared, indicating that the photoproduct **5**^2+^ was not stable upon prolonged irradiation, and/or that it further reacted with l-Asc or GSH, for example by coordination. Like in the previous experiment, the upconversion emission intensity increased in the first two hours of irradiation, revealing that the photoproduct was leaving the membrane of the liposome. As a control, the irradiation experiment under argon was repeated with **M34** liposomes, which did not contain photosensitizer **1** and hence could not produce TTA-UC. A slow evolution of the absorbance at 490 was observed, which is attributed to direct absorption of the red light by **4**^2+^ due to the low but non-zero absorption of the complex at 630 nm. Overall, these results demonstrate that light upconversion in air was efficient enough to greatly amplify the rate of photodissociation upon red light irradiation.

One of the remaining questions was whether the photoproduct obtained in air with antioxidants was identical to that obtained under argon. To answer this question, the irradiation experiments were repeated, but stopped after 60 min; the liposomes were removed from the solution using a centrifugal filtration, and a UV-vis absorption spectrum of the filtrate was recorded. When irradiation was performed under argon, the UV-vis absorption spectrum of the filtrate was identical to the absorption spectrum of **5**^2+^ (Figures S4 and S5), thereby confirming the photodissociation reaction of **4**^2+^ to **5**^2+^. When irradiation was performed in air and in presence of antioxidants, the absorption spectrum of the filtrate was very similar to that of **5**^2+^, suggesting that the same (or a similar) Ru-complex had been formed. As control, no absorption was detected for filtered solutions of **M134** liposomes kept in the dark under argon or with antioxidants. Finally, red light irradiation of **M134** was repeated at higher liposome concentration (20 mM DMPC), and mass spectra were recorded after centrifugal filtration, lyophilisation of the filtrate, and redissolving the residue in acetone (Figure S6). In case of irradiation under argon, the main peaks at *m*/*z* = 490.1 and 507.1 belonging to [Ru(bpy)_2_(OH_2_)(OH)]^+^ (MeCN) and [Ru(bpy)_2_(OH_2_)(OH)]^+^ (acetone), respectively, confirmed that **5**^2+^ had indeed formed. In the case of irradiation in air in presence of antioxidants, the sample only dissolved in methanol, which suggests that another Ru-complex had been formed. The mass spectrum showed peaks at *m*/*z* = 481.1 and 490.0 *m*/*z*, belonging to [Ru(bpy)_2_(OH_2_)(OH)]^+^ (MeOH) and [Ru(bpy)_2_(OH_2_)(OH)]^+^ (MeCN) (Figure S7). Although no signals were detected from a GSH complex, for example at *m*/*z* = 738.1 for [Ru(bpy)_2_(GS)(H_2_O)]^+^, it is still plausible that compound **5**^2+^ and products such as GSH adducts co-exist in the reaction mixture. Overall, these results show that the addition of GSH and l-Asc allowed the red light induced photoactivation cascade of TTA-UC, non-radiative energy transfer, and photosubstitution on ruthenium, to function in air.

### 2.4. At Which Tissue Depth Can Red-to-Blue TTA-UC Be Generated?

**Figure 4:**
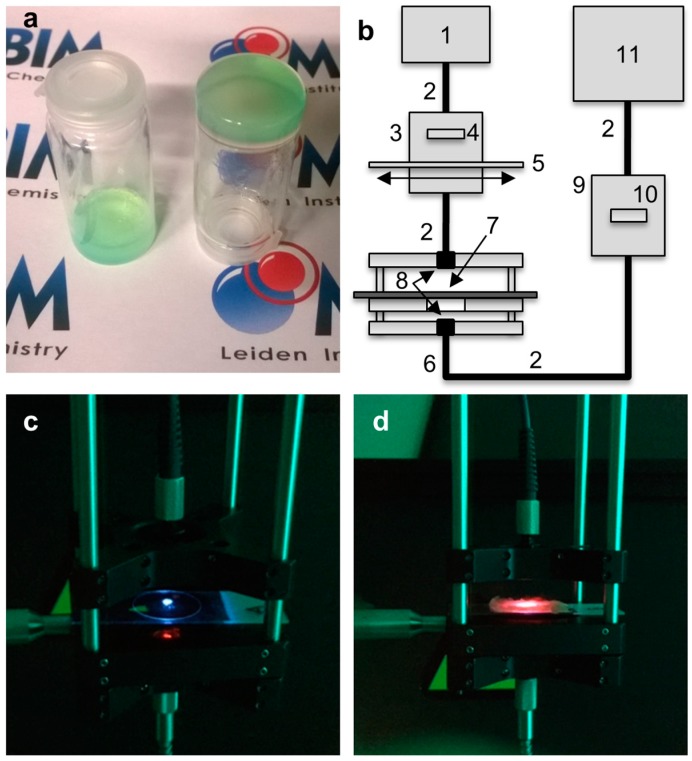
Upconversion spectroscopy with gellified **L12** liposomes buried under meat. (**a**) Photograph of bulk gellified **L12** liposomes in 0.5 wt % agarose gel in PBS containing 5 mM l-Asc and GSH; (**b**) Schematic representation of the experimental setup used for spectroscopy through meat: (1) 630 nm laser; (2) optical fibers; (3) filter holder for 630 nm band pass filter (4) and variable neutral density filter (5); (6) cage system with two oppositely facing cage plates, both fitted with a collimating lens (8), and a central sample-holding plate (7); (9) filter holder for a 630 nm notch filter (10); (11) CCD-spectrometer. Photographs of the cage spectroscopy setup with: the upconverting liposome gel only (**c**); and with 1 layer of pork fillet on top (**d**). Both photographs were taken with a 575 nm OD 4 short pass filter in front of the camera to block the excitation source.

A priori, the reason to use red-to-blue light upconversion for photoactivation chemotherapy is the increased excitation penetration depth of red light in tissues. However, up to now it remained unknown at which tissue depth red-to-blue TTA-UC can occur with liposomes. To answer this question in a model of healthy human tissue, an upconverting gel was first prepared by mixing **L12** liposomes ([DLPC] = 10 mM) with 0.5 wt % agarose and 5 mM l-Asc and GSH in PBS (see Figure 4a for a photograph of the gel in bulk). This gel was then deposited as a thin disk between two microscopy slides, and further covered with a variable stack of thin slices (1–2 mm thick) of raw chicken breast or pork fillet to mimic human tissue of different color and structure. Finally, a 630 nm laser beam (0.57 W/cm^2^) was directed perpendicularly to the meat, thus exciting the upconverting liposome gel at a power density that depended on the thickness of the meat layer above it. The emission spectrum of the upconverting gel was measured on the other side of the sample, thus in transmission mode, using a custom-made cage spectroscopy setup (Figure 4).

**Figure 5:**
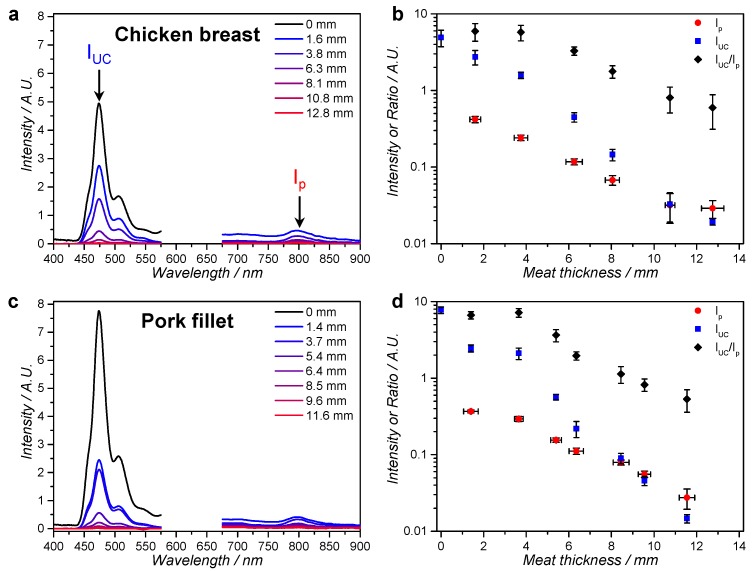
Emission spectroscopy of an upconverting liposome gel through layers of chicken breast or pork fillet. Emission spectra of gellified **L12** liposomes through layers of meat at room temperature as a function of: chicken breast thickness (**a**); or pork fillet thickness (**c**). Upconversion intensity (*I_UC_*, at 474 nm), phosphorescence intensity of compound **1** (*I_p_*) and the ratio of *I_UC_* and *I_p_* as a function of: chicken breast thickness (**b**) or pork fillet thickness (**d**). Conditions: 20 µL sample in a 25 µm × 25 mm diameter disk of **L12** liposomes ([DLPC] = 10 mM) in 0.5% wt % agarose gel, 5 mM l-Asc, and 5 mM GSH in PBS at room temperature, irradiated with 30 mW (3 mm spot, 0.57 W/cm^2^) 630 nm light through a variable number of meat slices. The spectra are cut off between 575 nm and 675 nm to omit the large excitation scatter peak.

The emission spectra for both meat types showed the typical phosphorescence of **1** at 800 nm and upconversion emission of **2** at 474 nm (Figure 5a,c). As could be expected, the intensity of the entire spectrum decreased as a function of meat thickness due to filtering and scattering of the excitation source by the meat. However, upconversion was still observable for 12.8 mm thick chicken and for 11.6 mm thick pork. Figure 5c,d shows the upconversion intensity (*I_UC_*), the phosphorescence of **1** (*I_p_*), and their ratio (*I_UC_*/*I_p_*) as a function of meat thickness. It can be clearly seen that from the third meat layer onwards, *I_UC_* decreases relatively faster than *I_p_*, i.e., *I_UC_*/*I_p_* decreases. This can be rationalized by the fact that TTA-UC is quadratically dependent on the excitation intensity below the intensity threshold for efficient upconversion (*I_th_*), while the phosphorescence is linearly dependent. *I_th_* was previously determined to be 0.05 W/cm^2^ for very similar red-to-blue TTA-UC in DOPC liposomes [42]. Considering that *I_UC_*/*I_p_* changes for the first time after three layers of meat, this must mean that the excitation intensity after three layers of meat has decreased from 0.57 W/cm^2^ to approximately 0.05 W/cm^2^). Overall, the results show that upconversion was generated beyond 10 mm penetration depth, but that, beyond 5 to 6 mm meat depth, the TTA-UC efficiency decreased rather quickly. Naturally, the depth at which the excitation intensity equals *I_th_* depends on the initial excitation intensity, so it was decided that a higher irradiance was needed to activate Ru-prodrugs through meat (see below).

### 2.5. Inducing TTA-UC and Drug Activation through Meat with DLPC Liposomes

**Figure 6:**
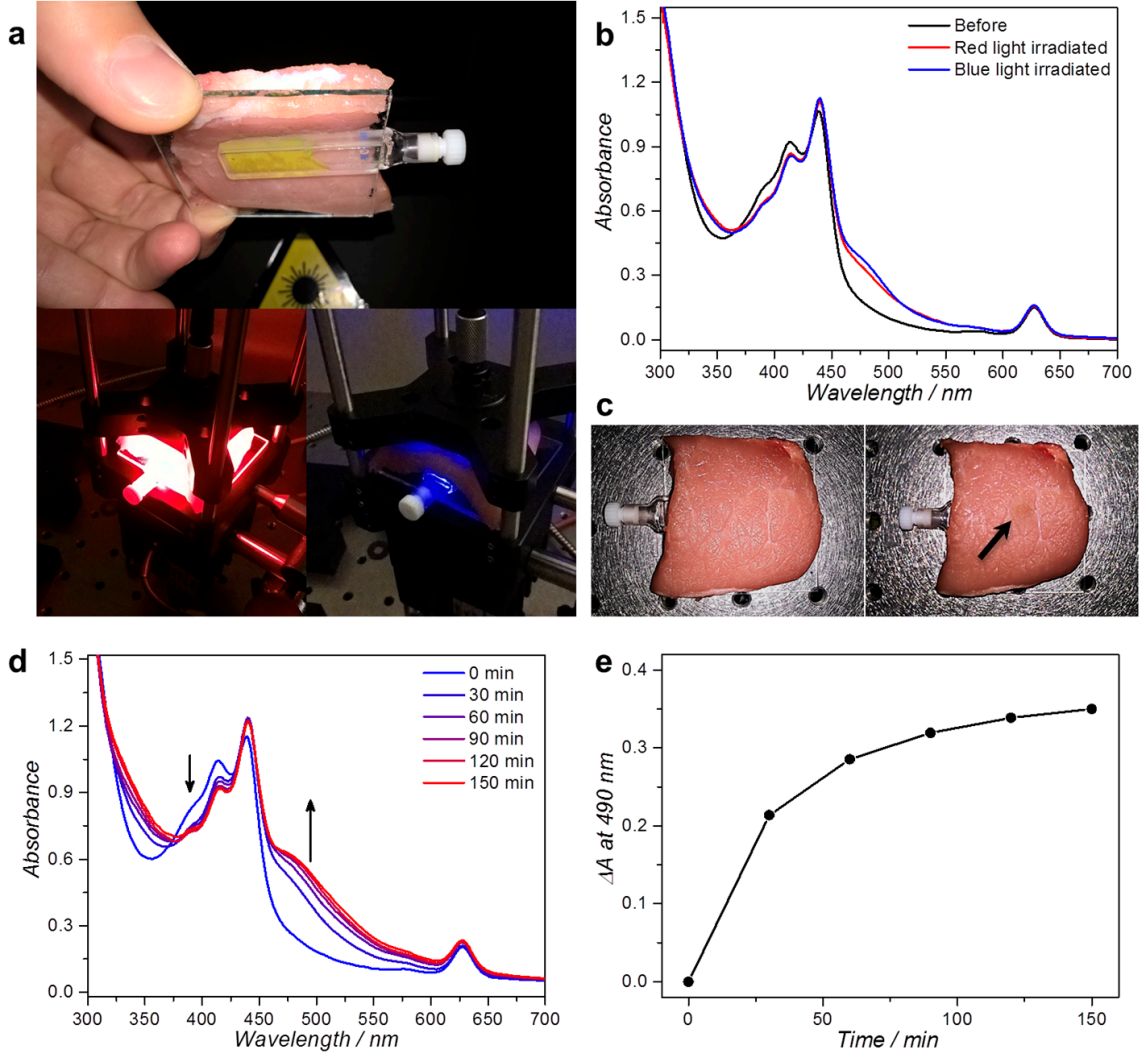
Irradiation of **L134** liposomes through a thick slice of pork fillet. (**a**) Photographs of the experimental setup used for red and blue light irradiation. The 2 mm cuvette holding 400 µL **L134** ([DLPC] = 10 mM) is covered with 7 mm pork fillet and irradiated for 2 h from above with a collimated 110 mW 630 or 450 nm beam (3 mm spot size, 1.6 W/cm^2^ intensity, 11.2 kJ/cm^2^ light dose); (**b**) UV-vis absorbance spectra of the sample before (black) and after irradiation with 450 nm (blue) or 630 nm light (red); (**c**) Photographs of the pork fillet after red (left) or blue light irradiation (right). Upon blue light irradiation, a clear “burn mark” was observed, as indicated with the arrow. UV-vis absorbance spectra (**d**); and the absorbance difference at 490 nm (**e**) as a function of irradiation time for **L134** liposomes irradiated through 7 mm pork fillet with 300 mW 630 nm light (4.2 W/cm^2^). Experiment performed once. Arrows indicate the evolution of the spectrum in time near the indicated wavelengths.

The real question in phototherapy, however, is whether it is really advantageous for the activation of Ru prodrugs to use red light and upconversion, or whether blue light, which penetrates less far into the tissue but triggers in a more straightforward manner the photosensitive compound, remains preferable. To compare both irradiation wavelengths, the photodissociation of **4**^2+^ into **5**^2+^ with red or blue light and **L134** liposomes was attempted through a layer of pork fillet (7 ± 0.5 mm) to simulate operation conditions. A 2 mm thick cuvette was filled with 400 µL **L134** liposomes ([DLPC] = 10 mM) in a buffer containing 10 mM l-Asc and GSH, and then it was placed under the layer of pork fillet. The sample was irradiated through the meat with either red or blue light for 2 h (Figure 6a) with equal light doses for both experiments (110 mW laser light, 3 mm spot size, 1.6 W/cm^2^, 11.2 kJ/cm^2^). The UV-vis absorption spectrum of the cuvette (without meat) was measured before and after irradiation to investigate how much of **4**^2+^ had been activated into **5**^2+^ (Figure 6b). Unexpectedly, the absorption band that appeared between 460 and 600 nm indicated that similarly small quantities of the aqua species **5**^2+^ (compare Figure 3a and Figure 6b) were obtained for both excitation wavelengths.

This result can be interpreted by considering the two different photochemical pathways of activation. In the case of blue light, the light intensity quickly diminishes with penetration depth, but the photons that do reach the sample have a high chance of being absorbed due to the high absorbance of **4**^2+^ at 450 nm (*ε*_450_ ≈ 6000 M^−1^·cm^−1^, [**4**^2+^] = 0.40 mM; $A_{450}^{4^{2+}}$ ≈ 0.5 for 2 mm path length). In addition, upon blue light absorption, the complex is directly activated without intervention of TTA-UC, i.e., the chance is comparatively high that absorption of a blue photon causes photodissociation. In the case of red light, the light intensity diminishes much less quickly with meat thickness, i.e., more light reaches the cuvette for a given thickness than with blue light. However, the overall activation efficiency is the product of the upconversion quantum yield (*Φ_UC_*), of the energy transfer efficiency from annihilator **3** to **4**^2+^ (*E_ET_*), and of the photodissociation quantum yield of the ruthenium complex (*Φ_Ru_*), which leads overall to a ~20 times lower activation efficiency [26]. In addition, the absorbance of compound **1** at 630 nm was comparatively low ($A_{630}^{1}$ = 0.15), leading to less efficient use of the red light that permeates at higher intensities through the meat. Last but not least, at 1.6 W/cm^2^ 630 nm excitation intensity, it is likely that *Φ_UC_* is lower than its maximum value, because the excitation intensity likely drops to *I_th_* or below (~0.05 W/cm^2^). Thus, even though **L134** liposomes are clearly more responsive to blue light, the data show that the overall activation rate with red light was approximately just as high as with blue light at the same power.

However, one observation was still missing: the state of the meat after 2 h irradiation. Visual inspection revealed that while red light had caused no apparent damage, blue light had clearly burned the meat considerably at the irradiation spot (Figure 6c). This result is consistent with in vitro data of our group that blue light is much more harmful for cells than green or red light [43]. In operation conditions, red light will not harm the superficial tissues, which is much more favorable than blue light. Tissue burns are also a great issue for lanthanoid-based upconversion nanoparticles (UCNPs). For instance, it has been shown by Wu et al. that irradiation of chicken meat with 980 nm laser light for 20 min at ≥5 W/cm^2^ intensity causes significant burn marks. In the case of red-to-blue TTA-UC, our data show that upconversion-mediated drug activation is possible at relatively low power (110 mW) without any visible tissue ablation.

In the previous experiment, prodrug activation was limited to 10%–20% of the initially available amount of Ru compound notably because our blue laser could not provide higher light intensities. The red laser did not have such power limitations. To finally prove that it is possible to fully activate the ruthenium compound through meat, a similar red light irradiation experiment was repeated using higher intensity red light (300 mW, 4.2 W/cm^2^ power density), and the absorption spectrum of the liposome solution was measured every 30 min (Figure 6d,e). The absorption band for **5**^2+^ between 460 and 600 nm reached a plateau after approximately 2 h of irradiation, which indicated that full activation could indeed be obtained provided the red light intensity and dose was high enough. Even at such power densities of red light no visible signs of irradiation damage or tissue burn were observed. In conclusion, our data show that activation of complex **4**^2+^ by a combination of red light irradiation and TTA-UC was complete even through a 7 mm thick slice of pork fillet and under air. At such red light intensities, activation is not necessarily much faster than using blue light, but the compound is activated with much less tissue damage for any healthy tissue that may be surrounding the tumor.

## 3. Materials and Methods

### 3.1. General

Palladium tetraphenyltetrabenzoporphyrin (**1**) was purchased from Frontier Scientific, Inc. (Logan, UT, USA). Perylene (**2**) was purchased from Sigma-Aldrich Chemie BV (Zwijndrecht, The Netherlands). The synthesis of 2,5,8,11-tetra(tert-butyl)perylene (compound **3**) is described elsewhere [25]. Sodium *N*-(carbonyl-methoxypolyethylene glycol-2000)-1,2-distearoyl-*sn*-glycero-3-phosphoethanolamine (DSPE-mPEG-2000), 1,2-dilauroyl-*sn*-glycero-3-phosphocholine (DLPC), and 1,2-dimyristoyl-*sn*-glycero-3-phosphocholine (DMPC) were purchased from Lipoid GmbH (Ludwigshafen, Germany) and stored at −18 °C. Dulbecco’s phosphate buffered saline (PBS) was purchased from Sigma Aldrich and had a formulation of 8 g/L NaCl, 0.2 g/L KCl, 0.2 g/L KH_2_PO_4_, and 1.15 g/L K_2_HPO_4_ with a pH of 7.1–7.5. Antioxidant supplemented PBS was prepared by dissolving l-ascorbic acid and/or glutathione in PBS and neutralizing with sodium hydroxide to pH 7.0–7.6. Other chemicals were purchased from major chemical suppliers and used as received. Data was processed with Origin Pro and Microsoft Excel software. Photodissociation experiments using blue light were recorded in a Varian Cary 50 spectrometer (Agilent Technologies, Amstelveen, The Netherlands) equipped with a Cary Single Cell Peltier for temperature control.

### 3.2. Synthesis of 1,3-Bis(methylthio)-2-dodecyloxypropane—Compound ***6***

To a stirred suspension of NaH (222 mg, 5.55 mmol) in dry and deoxygenated THF (5 mL) under Ar atmosphere was added 1,3-bis(methylthio)-2-propanol (0.25 mL, 1.85 mmol). The mixture was stirred at RT for 10 min, after which 1-bromododecane (0.5 mL, 2.13 mmol) was added dropwise, resulting in a light-brown suspension. The reaction mixture was heated to reflux for 20 h. Hereafter, the mixture was concentrated to 1–2 mL, Et_2_O (40 mL) was added, and the resulting mixture was washed with brine (40 mL), 1 M aq. NH_4_Cl (2 × 40 mL) and brine (40 mL). The organic layer was dried with MgSO_4_ and the solvent was removed by rotary evaporation. After column chromatography (SiO_2_, petroleum ether 40–60:DCM (2:1) to neat DCM) the title compound was obtained as a light-yellow oil (250 mg, 0.78 mmol, 42%). TLC: *R*_f_ = 0.9 (DCM). ^1^H-NMR (300 MHz, δ in CDCl_3_): 3.58 (p, *J* = 5.8 Hz, 1H, H3), 3.51 (t, *J* = 6.6 Hz, 2H, H1′), 2.74 (ddd, *J* = 18.9, 13.6, 5.8 Hz, 4H, H2 + H4), 2.16 (s, 6H, H1 + H5), 1.65–1.51 (m, 2H, H2′), 1.42–1.18 (m, 18H, H3′-H11′), 0.88 (t, *J* = 6.5 Hz, 3H, H12′); ^13^C-NMR (75 MHz, δ in CDCl_3_): 79.3, 70.3, 37.7, 32.1, 30.2, 29.8, 29.8, 29.8, 29.7, 29.6, 29.5, 26.3, 22.8, 16.9, 14.3; ESI-MS *m*/*z* exp. (calcd.): 135.1 (135.0, [M − C_12_H_25_O]^+^), 208.0 (208.2, [C_12_H_25_O + Na]^+^), 321.2 (321.2, [M + H]^+^).

### 3.3. Synthesis of ***4**(PF_6_)_2_*

**Scheme 2:**
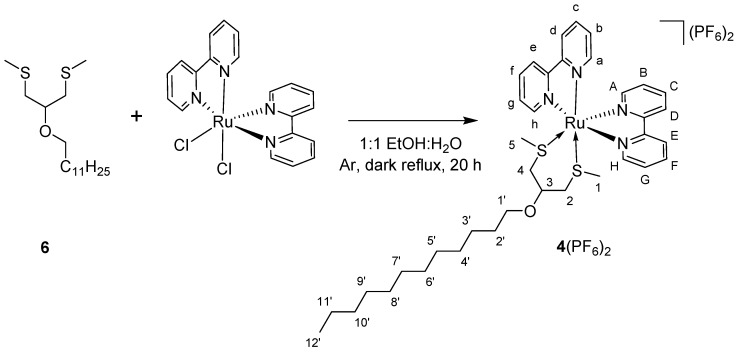
Synthesis of **4**(PF_6_)_2_ from compound **6** and *cis*-Λ/Δ-[Ru(bpy)_2_Cl_2_].

A mixture of compound **6** (66 mg, 0.206 mmol) and *cis*-Λ/Δ-[Ru(bpy)_2_Cl_2_] (50 mg, 0.103 mmol) under Ar atmosphere was dissolved in a 1:1 mixture of EtOH and H_2_O (10 mL) and heated to reflux in the dark for 20 h. Hereafter, the reaction mixture was cooled to room temperature, and the solvent was removed in vacuo. The reaction mixture was poured onto 30 mL of sat. aq. KPF_6_, extracted with DCM (4 × 20 mL), and concentrated by rotary evaporation. Removal of the excess ligand was done by centrifugal washing with diethyl ether (2 × 12 mL, 2800× *g*). *cis*-Λ/Δ-[Ru(bpy)_2_(**6**)](PF6)_2_ (compound **4**(PF_6_)_2_ Scheme 2) was obtained as an orange powder in 45% yield (47 mg, 0.046 mmol). ^1^H-NMR (400 MHz, δ in CD_3_CN): 9.62 (d, *J* = 5.3 Hz, 1H, H_H_), 9.17 (d, *J* = 5.3 Hz, 1H, H_h_), 8.52 (t, *J* = 8.7 Hz, 2H, H_e_ + H_E_), 8.38 (dd, *J* = 8.2, 2.8 Hz, 2H, H_d_ + H_D_), 8.33–8.24 (m, 2H, H_f_ + H_F_), 8.03–7.96 (m, 2H, H_c_ + H_C_), 7.93–7.85 (m, 2H, H_g_ + H_G_), 7.49 (d, *J* = 5.1 Hz, 1H, H_A_), 7.43 (d, *J* = 5.1 Hz, 1H, H_a_), 7.36–7.26 (m, 2H, H_b_ + H_B_), 4.37 (s, 1H, H3), 3.67 (dt, *J* = 9.2, 6.5 Hz, 1H, H1′), 3.49 (dt, *J* = 9.2, 6.5 Hz, 1H, H1′), 3.20 (dd, *J* = 13.2, 6.2 Hz, 1H, H4), 3.12 (dd, *J* = 13.9, 2.7 Hz, 1H, H2), 2.95 (s, 1H, H2), 2.61 (dd, *J* = 13.1, 1.6 Hz, 1H, H4), 1.66–1.54 (m, 2H, H2′), 1.43 (s, 3H, H1), 1.40–1.21 (m, 18H, H3′-11′), 1.08 (s, 3H, H5), 0.88 (d, *J* = 6.8 Hz, 3H, H12′); ^13^C-NMR (100 MHz, δ in CD_3_CN): 158.6 (C_q_), 158.5 (C_q_), 157.3 (C_q_), 157.2 (C_q_), 154.6 (C_h_), 153.9 (C_H_), 152.2 (C_a_), 151.9 (C_A_), 139.8 (C_c_ + C_C_ + C_f_ + C_F_), 129.7 (C_g_), 128.7 (C_b_ + C_B_), 128.6 (C_G_), 125.9 (C_e_), 125.8 (C_E_), 125.2 (C_d_), 125.1 (C_D_), 74.2 (C3), 70.4 (C1′), 38.0 (C4), 37.2 (C2), 32.6, 30.5, 30.3, 30.3, 30.0, 26.8, 23.4, 20.9 (all C2′-11′), 18.5 (C1), 16.0 (C5), 14.4 (C12′); HR-MS *m*/*z* exp. (calcd.): 367.1310 (367.1312, [M − 2PF_6_]^2+^); UV-Vis: λ_max_ (ε in L/mol∙cm) in CH_3_CN: 415 nm (6170); Elemental analysis for C_37_H_52_F_12_N_4_OP_2_RuS_2_: (calcd.): C, 43.40; H, 5.12; N, 5.47; (exp.): C, 43.38; H, 5.15; N, 5.49.

### 3.4. Liposome Preparation

All liposome formulations were prepared by the classical hydration-extrusion method. As an example, the preparation of **M134** is described here. Aliquots of chloroform stock solutions containing the liposome constituents were added together in a flask to obtain a solution with 5.0 µmol DMPC, 0.20 µmol DSPE-mPEG-2000, 2.5 nmol **1**, 25 nmol **3**, and 0.20 µmol **4**(PF_6_)_2_. The organic solvent was removed by rotary evaporation and subsequently under high vacuum for at least 30 min to create a lipid film. One milliliter PBS buffer (with or without l-ascorbic acid and/or glutathione) was added and the lipid film was hydrated by 4 cycles of freezing the flask in liquid nitrogen and thawing in warm water (50 °C). The resulting dispersion was extruded through a Whatman Nuclepore 0.2 μm polycarbonate filter at 40–50 °C at least 11 times using a mini-extruder from Avanti Polar Lipids, Inc. (Alabaster, AL, USA). The number of extrusions was always odd to prevent any unextruded material ending up in the final liposome sample. The extrusion filter remained practically colorless after extrusion, suggesting near-complete inclusion of the chromophoric compounds in the lipid bilayer. Liposomes were stored in the dark at 4 °C and used within 7 days. The average hydrodynamic liposome size and polydispersity index (PDI) were measured with a Zetasizer Nano-S machine from Malvern Instruments (Malvern, UK), operating at a wavelength of 632 nm. The hydrodynamic diameter and polydispersity index (PDI) were typically 130−170 nm and 0.1, respectively.

### 3.5. Upconversion Emission Spectroscopy and Photodissociation Experiments with Red Light

Upconversion emission spectroscopy and photodissociation experiments were conducted in a custom-built setup (Figure S8). All optical parts were connected with FC-UVxxx-2 (xxx = 200, 400, or 600) optical fibers from Avantes (Apeldoorn, The Netherlands), with a diameter of 200–600 μm, respectively, and that were suitable for the UV-Vis range (200–800 nm). In the case of deoxygenation by argon bubbling, 1.5 mL of the diluted liposome sample was bubbled for at least 30 min in an external ice-cooled pear-shaped flask. After this period, bubbling was stopped while maintaining the argon flow, and the sample was warmed in a water bath of approximately 40 °C for 10 min. Then, the sample was transferred by means of cannulation with argon pressure to a 111-OS macro fluorescence cuvette from Hellma (Müllheim, Germany) in a CUV-UV/VIS-TC temperature-controlled cuvette holder from Avantes. The sample was allowed to equilibrate at 310 K for an additional 10 min while stirring. The sample was held under argon atmosphere at a constant temperature of 310 K and irradiated for 4 h from the side with a 630 nm laser light beam from a clinical grade Diomed 630 nm PDT laser. The 630 nm light was filtered through a FB630-10, 630 nm band pass filter (Thorlabs, Dachau/Munich, Germany) put between the laser and the sample. The excitation power was controlled using a NDL-25C-4 variable neutral density filter (Thorlabs), and measured using a S310C thermal sensor connected to a PM100USB power meter (Thorlabs). The laser was collimated to a beam of 4 mm diameter with a power of 10 or 150 mW (0.080 or 1.2 W/cm^2^ intensity); in such conditions, a cylinder of approximately 0.13 cm^3^ was simultaneously excited by the laser (8% of the total sample volume). UV-Vis absorption spectra were measured using an Avalight-DHc halogen-deuterium lamp (Avantes) as light source and a 2048L StarLine spectrometer (Avantes) as detector, both connected to the cuvette holder at a 180° angle and both at a 90° angle with respect to the red laser irradiation direction. The filter holder between cuvette holder and detector was in a position without a filter (Figure S8, item 8). Luminescence emission spectra were measured using the same detector but with the UV-Vis light source switched off. To visualize the spectrum from 450 nm to 900 nm, while blocking the red excitation light, a Thorlabs NF-633 notch filter was used in the variable filter holder. For photodissociation experiments, a UV-Vis absorption and an emission spectra were measured every 15 min; each time the emission spectra were measured first by switching the filter holder to the appropriate position, then the laser was switched off, the halogen-deuterium lamp was turned on, the filter holder was switched to an open position, a UV-Vis absorption spectrum was recorded, the halogen-deuterium lamp was switched off, and the laser was switched on again. Each UV-Vis measurement took approximately 15 s in total. All spectra were recorded with Avasoft software from Avantes and further processed with Microsoft Office Excel 2010 and Origin Pro 9.1 software (OriginLab Corp., Northampton, MA, USA).

### 3.6. Upconversion Emission Spectroscopy with ***L12*** Liposomes and Meat

**L12** liposomes were prepared according to Section 3.4 ([DLPC] = 20.0 mM). To prepare a liposome hydrogel and to allow upconversion in air, this solution was heated to 55 °C and 1:1 *v*/*v* mixed with a solution at 55 °C containing 1 wt % agarose, 10 mM sodium l-ascorbate and 10 mM sodium glutathionate (pH 7.3). Twenty microliters of this mixture was pipetted with a warm pipet on a warm 1 mm thick microscopy slide (Menzel-Gläser Superfrost, 76 × 26 mm) and immediately covered with a round 25 mm diameter microscopy coverslip (VWR, thickness no. 1). Upon cooling to room temperature, this procedure produced a thin gel slice with ~25 µm thickness. On top of the coverslips, thin slices of chicken breast fillet or pork fillet were layered (1–2 mm thick each, measured for each slice with a caliper) up to ~13 mm thick. The entire sample construct was allowed to reach room temperature (20 °C) before measurement in a custom-build spectroscopy setup (Figure 4). A cage with an open sample space was constructed with single collimating lenses (Avantes COL-UV/VIS) on both sides for excitation (from the top) and detection (from the bottom) connected with FC-UV400-2 or FC-UV600-2 optical fibers (Avantes). The excitation lens was connected to a clinical grade Diomed 630 nm PDT laser set to 30 mW (3 mm beam, 0.42 W/cm^2^) using a PM100 USB power meter and S310C detector (Thorlabs). Between the laser and the excitation lens, a filter holder was placed with a FB630-10 band pass filter and a NDL-25C-4 variable neutral density filter (Thorlabs). The detection lens was connected to a 2048L StarLine spectrometer (Avantes); between the spectrometer and detection lens, a filter holder with a NF-633 notch filter was placed. For each meat layer thickness, the spectrum was taken at 5 different sample locations, and the spectrum was averaged. Then, a new layer of meat was carefully placed on top and the measurements were repeated until a thickness of ~13 mm was achieved.

### 3.7. Photodissociation Experiments with Meat

For photodissociation experiments with **L134** liposomes and meat, the sample was prepared as described in Section 3.4 ([DLPC] = 10 mM). Four hundred microliters sample was placed in a 2 mm thick cuvette and sandwiched between a 5 cm × 5 cm glass plate and a 5 cm × 5 cm slice of pork fillet (Albert Heijn, Zaandam, The Netherlands; “AH Filetlapjes à la minute naturel”), 7 ± 0.5 mm thick. This meat-sample construct was placed in the same cage setup as described in Section 3.6, but the laser was now directly coupled to the excitation lens and the FB630-10 band pass filter was now placed directly between the excitation lens and the sample (see Figure 6). The detection lens and spectrometer were left out. To compare blue and light irradiation, the meat-sample construct was irradiated from the top with 110 mW of either 450 nm or 630 nm laser light in a 3 mm collimated beam (1.6 W/cm^2^). In addition, the experiment was repeated with 300 mW 630 nm light (4.2 W/cm^2^).

## 4. Conclusions

In this article, the biological applicability of upconverting liposomes for the activation of ruthenium-based anticancer prodrugs with red light was assessed. Using an antioxidant-supplemented buffer and air it was possible to obtain enough blue light upon red light irradiation of the liposomes to activate a Ru compound (**4**^2+^) located in the membrane containing the upconverting dyes. To approach the practical conditions of a phototherapeutic operation, it was first determined that upconverted blue light can still be generated by TTA-UC through more than 10 mm of chicken or pig meat. When filtering the excitation source with a 7 mm thick pork fillet slice, Ru photodissociation could be triggered at comparable rates either using blue light or red light (1.6 W/cm^2^) and red-to-blue upconverting liposomes. However, significant tissue damage was obtained with blue light, whereas the same dose of red light did not lead to any observable tissue damage. It was then possible to increase the power density to ~4 W/cm^2^ to obtain full activation of the Ru compound with red light. Overall, these results pave the way for TTA-UC mediated photoactivation of photochemotherapeutic drugs in a biological context.

## Figures and Tables

**Table 1:** Summary of liposome formulations used in this chapter. Liposomes with designation L or M are made with DLPC or DMPC as main lipid, respectively. Final concentrations are given as bulk concentrations (in mM or μM).

Code	[DMPC]	[DLPC]	[DSPE-mPEG-2000]	[1]	[2]	[3]	[4^2+^]
	mM	mM	mM	μM	μM	μM	μM
**L12**		5.0	0.20	2.5	25		
**L134**		5.0	0.20	2.5		50	200
**M134**	5.0		0.20	2.5		50	200
**M34**	5.0		0.20			50	200

## Supplementary Materials

Supplementary materials can be accessed at: http://www.mdpi.com/1420-3049/21/11/1460/s1.
