# Supplementary material for: Red Light Activation of Ru(II) Polypyridyl Prodrugs via Triplet-Triplet Annihilation Upconversion: Feasibility in Air and through Meat
Source: Molecules. 2016 Nov 1;21(11):1460. doi: 10.3390/molecules21111460 (PMC6273732; doi:10.3390/molecules21111460)
Supplement: Supplementary file 1 [file molecules-21-01460-s001.pdf]

Supplementary Materials: Red Light Activation of Ru(II) Polypyridyl Prodrugs via Triplet-Triplet Annihilation Upconversion: Feasibility in Air and Through Meat

Sven H. C. Askes, Michael S. Meijer, Tessel Bouwens, Iris Landman and Sylvestre Bonnet

1. Mechanism of TTA-UC

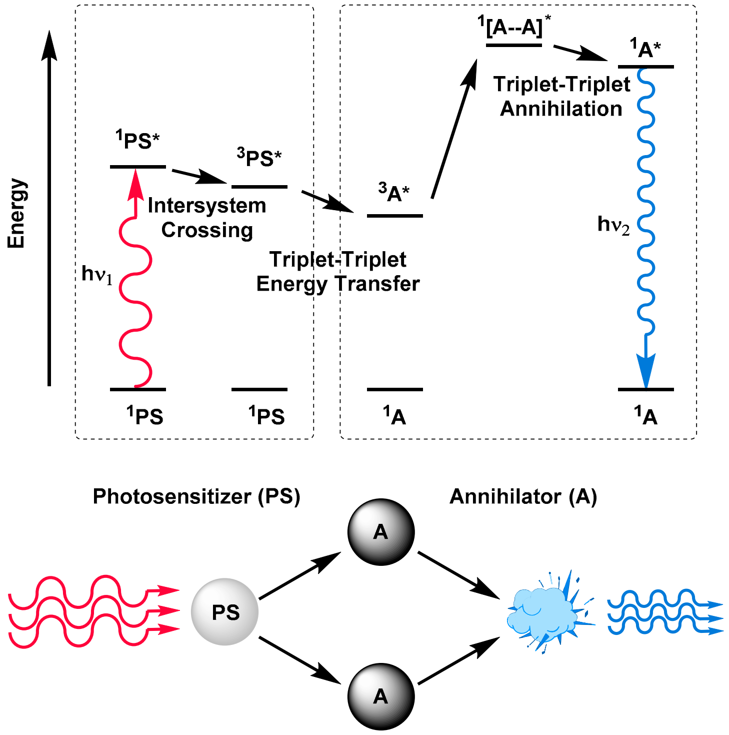

Figure S1. Top: Jablonski diagram of the photophysical processes involved in TTA-UC. Asterisks (*) indicate excited states. **Bottom**: scheme representing the generation of blue light by collision of two excited annihilator molecules.

2. Evaluation of Lag-Time with L12 Liposomes and Anti-Oxidants

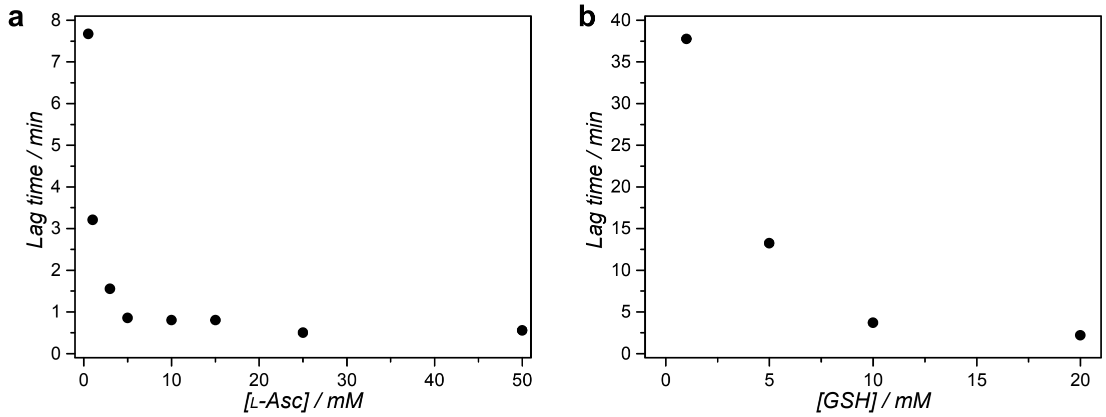

Figure S2. Lag-time as a function of the concentrations [L-Asc] (**a**) or [GSH] (**b**).

3. Overlap between Emission of 3 and Absorption of 4^{2+}

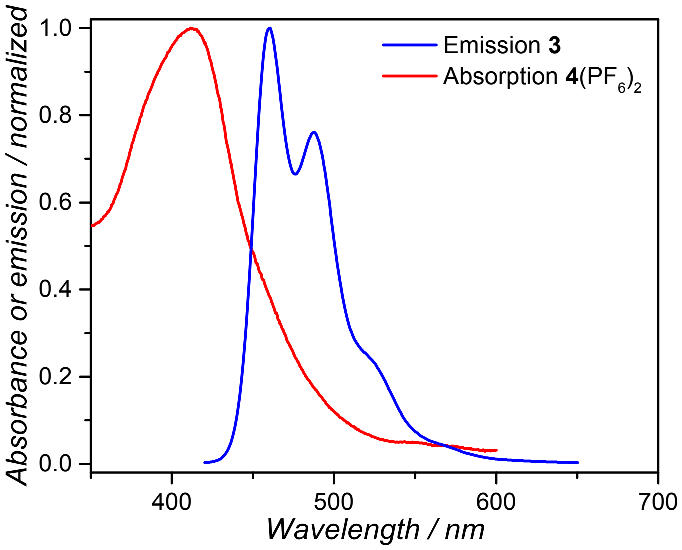

Figure S3. Overlap between normalized absorption spectrum of Ru-complex **4**$^{2+}$ (red) and the normalized emission spectrum of compound **3** (blue).

4. Photosubstitution of Ru-Complex 4^{2+} with Blue Light

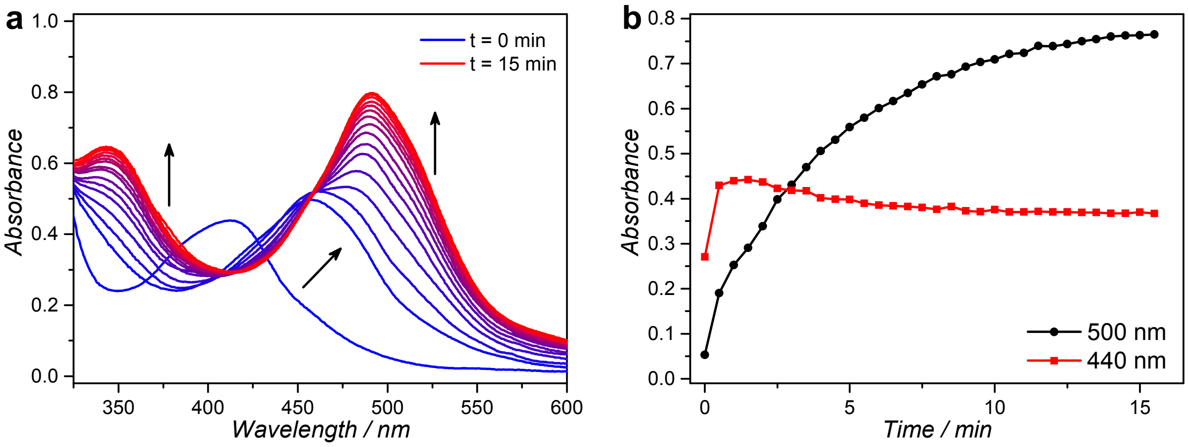

Figure S4. Time-dependent UV-vis absorption spectroscopy of complex **4**$^{2+}$ (0.07 mM) during blue light irradiation (450 nm, photon flux 0.17 μEinstein.s^{-1}) in water at 25 °C. (**a**) Absorption spectra, recorded every 1 min (blue to red evolution). Arrows indicate evolution of the spectrum in time. (**b**) Evolution of the absorbance at 440 nm (red squares) and 500 nm (black circles). The water was deoxygenated for 10 min by bubbling with argon and the solution was kept under an argon atmosphere during spectroscopy. The spectral evolution upon reaction of **4**$^{2+}$ to **5**$^{2+}$ shows that the photoreaction proceeds via two distinct steps: it is proposed that the first step is fast and involves the release of one of the thioether-ruthenium bonds (see how the spectrum changes in the first minute), and the second step is slower and involves the release of the other thioether-ruthenium bond.

5. UV-Vis Spectroscopy and Mass Spectrometry after Red-Light Irradiation of M134 Liposomes

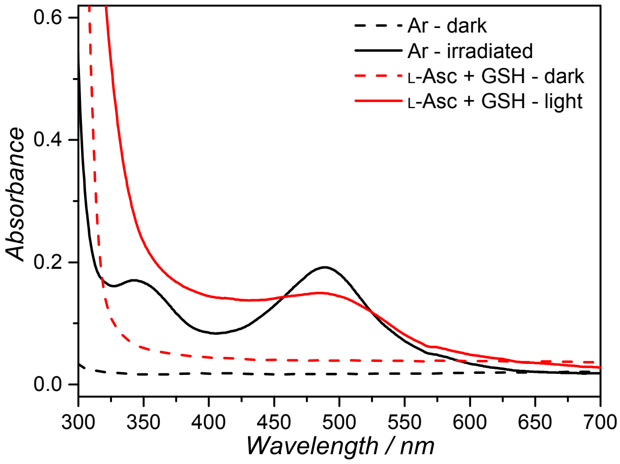

Figure S5. UV-vis absorption spectra of filtered solutions of red-light irradiated **M134** liposomes ([DMPC] = 1 mM) under argon (solid black) and in air in presence of 10 mM L-Asc and GSH (solid red). Irradiation was done for 60 min with 2 mL sample volume, 150 mW 630 nm light (1.2 W/cm^2 intensity, 4.3 kJ/cm^2), and at 37 °C, and then the solution was filtered with a centrifuge filter (MWCO = 100,000 Da); the UV-vis absorption spectrum of the filtrate is shown here. As controls, samples were kept in the dark and filtered in the same way (dashed lines): these spectra show neither absorption of the upconversion compounds **1** and **3**, nor that of the Ru-complex $\mathbf{4}^{2+}$, which indicates that no Ru photosubstitution has taken place and that the liposomes remain in the filter.

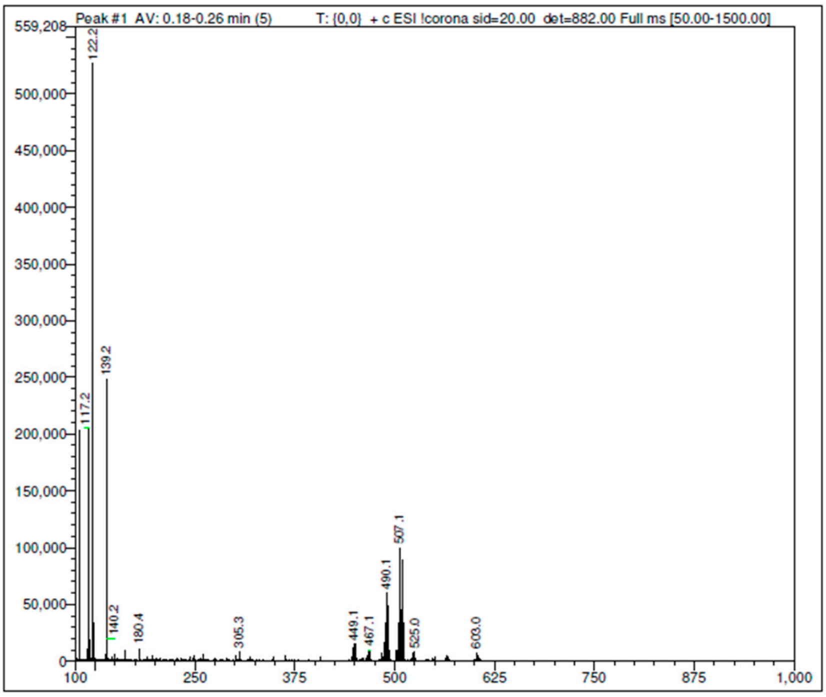

Figure S6. Mass spectrometry after red light irradiation of **M134** liposomes (DMPC = 20 mM). After 60 min irradiation with 150 mW 630 nm light (1.2 W/cm^2) at 37 °C under argon, the liposome solution was filtered with a centrifuge filter (MWCO = 100,000 Da), the filtrate was lyophilized and redissolved in a minimal amount of acetone. Mass spectrometer eluens was 50:50 *v*/*v* MeCN:H_2O. Attribution of main peaks in *m*/*z* (calculated): 449.1 $[Ru(bpy)_2Cl]^+$ (449.0); 467.1 $[Ru(bpy)_2Cl(OH_2)]^+$ (467.0); 490.1 $[Ru(bpy)_2(OH_2)(OH)]^+$(MeCN) (490.1); 507.1 $[Ru(bpy)_2(acetone)Cl]^+$ (507.1) or $[Ru(bpy)_2(OH_2)(OH)]^+$(acetone) (507.1).

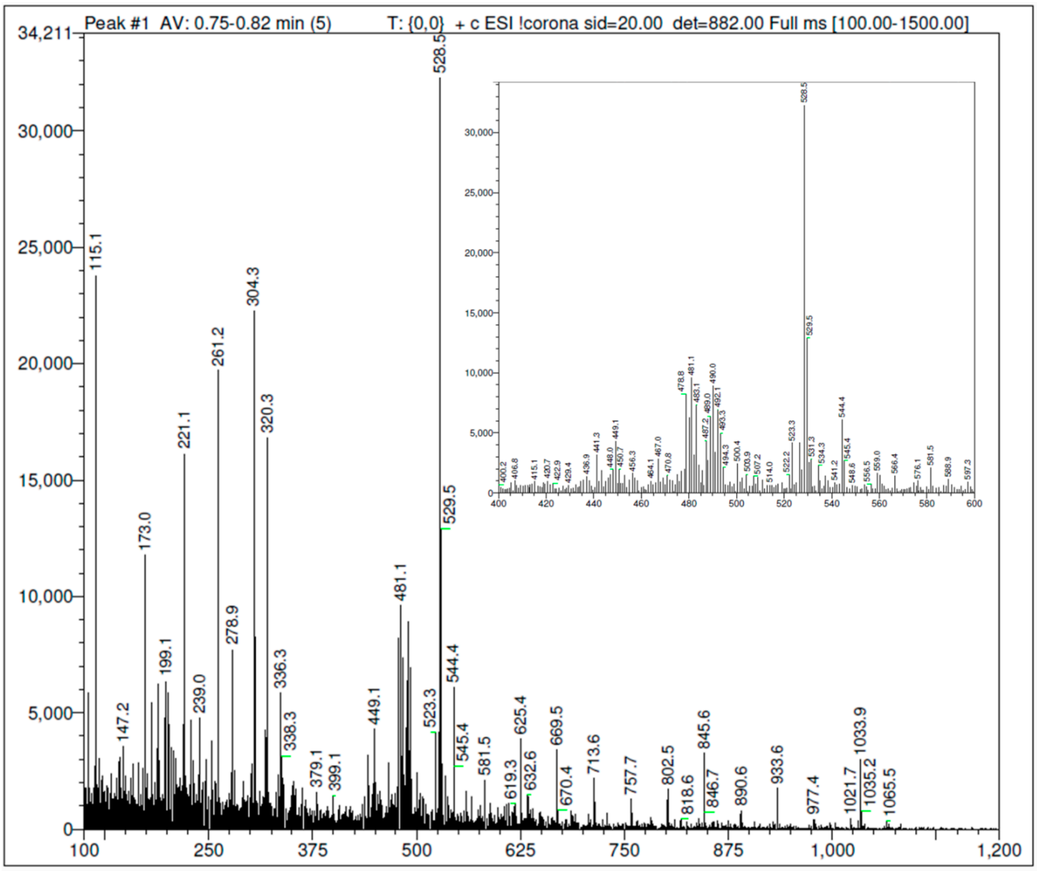

Figure S7. Mass spectrometry after red light irradiation of **M134** liposomes (DMPC = 20 mM) in presence of 10 mM L-Asc and GSH. After 60 min irradiation with 150 mW 630 nm light (1.2 W/cm^2) at 37 °C in air, the liposome solution was filtered with a centrifuge filter (MWCO = 100,000 Da), the filtrate was lyophilized and redissolved in a minimal amount of methanol. Mass spectrometer eluens was 50:50 *v*/*v* MeCN:H_2O. Attribution of main peaks in *m*/*z* (calculated): 481.1 [Ru(bpy)$_2$(MeOH)Cl]$^+$ (481.0) or [Ru(bpy)$_2$(OH$_2$)(OH)]$^+$(MeOH) (481.1); 490.0 [Ru(bpy)$_2$(OH$_2$)(OH)]$^+$(MeCN) (490.1). The rest of the signals do not contain a ruthenium isotope pattern.

6. Photodissociation Experiments Using Red Light

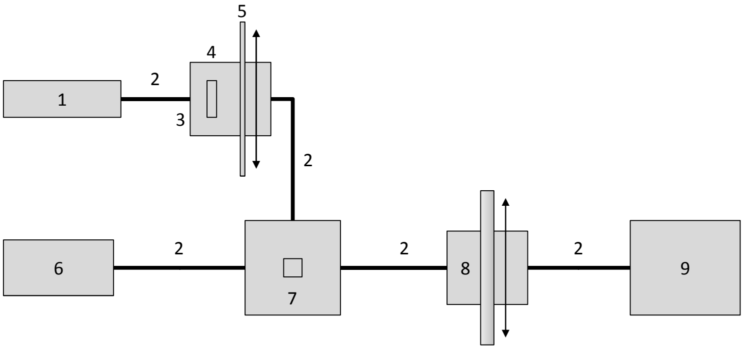

Figure S8. Setup used for photosubstitution experiments using red light. Legend: (**1**) 630 nm laser source, (**2**) optical fibers, (**3**) filter holder, (**4**) 630 nm band pass filter, (**5**) variable neutral density filter that can be installed or removed, (**6**) halogen-deuterium light source for UV-Vis absorption spectroscopy, (**7**) temperature controlled cuvette holder, (**8**) variable filter holder, and (**9**) CCD spectrometer.
